# Dysregulation of the inside-out signaling pathway in CNS-infiltrated pediatric T-cell acute lymphoblastic leukemia

**DOI:** 10.21203/rs.3.rs-9944183/v1

**Published:** 2026-07-05

**Authors:** Frida Holm, Sabina Enlund, Konstantinos Papadakis, Jacob Short, Katja Pokrovskaja Tamm, Ola Hermanson, Anna Nilsson, Qingfei Jiang

**Affiliations:** Karolinska Institutet; Karolinska Institutet; Karolinska Institutet; Karolinska Institutet; Karolinska Institutet; Karolinska Institutet; University of California, San Diego

## Abstract

A major obstacle to improving treatment efficacy and long-term survival in children with T-cell acute lymphoblastic leukemia (T-ALL) is the limited understanding of how leukemia cells infiltrate the central nervous system (CNS). By migrating to the CNS, leukemia cells can evade systemic therapy, contributing to disease progression and relapse. A better understanding of the mechanisms driving CNS infiltration in T-ALL could improve both diagnostic strategies and therapeutic interventions, thereby reducing the risk of CNS-originated relapse. One potential mechanism involves T-cell receptor (TCR)-mediated inside-out signaling, a pathway that regulates migration in normal T-cells and may be hijacked by leukemia cells invading the CNS. To investigate this possibility, we examined the role of this pathway in CNS-infiltrated pediatric T-ALL. RNA-sequencing analysis revealed enrichment of genes associated with the TCR signaling pathway, including adaptor protein SKAP1, a key component of inside-out signaling, in CNS-infiltrated and CNS-relapsed pediatric T-ALL samples. In addition, T-ALL cells exposed to methotrexate and co-cultured with meningeal cells exhibited alterations in signaling events downstream of the TCR. Knockdown of SKAP1 further resulted in reduced viability and proliferation of T-ALL cells. Collectively, these findings suggest that disruption of inside-out signaling may be characteristic for patients with CNS disease and could aid in providing new insights into the transcriptional programs underlying CNS infiltration in pediatric T-ALL.

## INTRODUCTION

T-cell acute lymphoblastic leukemia (T-ALL) is an aggressive hematological malignancy characterized by aberrant proliferation of immature lymphocytes (1). The pathology of T-ALL is complex, largely due to the heterogeneity of patient genetics and chromosomal abnormalities (2). Over the past decades, the 5-year overall survival for children diagnosed with T-ALL has increased immensely, reaching approximately 90% (2, 3). However, a substantial risk of relapse remains, partly due to migrating leukemia cells that enter and reside in the central nervous system (CNS). Leukemia cells that infiltrate the CNS can evade systemic chemotherapy, leading to the implementation of intrathecal methotrexate (MTX) in routine care (4, 5). CNS-directed therapy is administered to all children with T-ALL, which has greatly improved the survival outcomes, but can cause severe neurotoxicity and late effects (5–7). In order to enhance current treatment options and minimize adverse effects, we need to advance our mechanistic understanding of how leukemia cells infiltrate and survive in the CNS (4).

The main site of leukemia infiltration is the meninges—the protective layers that surround the brain and spinal cord—which contain the cerebrospinal fluid (CSF) (5, 8). Potential entry routes for leukemia cells into the CNS include the blood-leptomeningeal barrier and the blood-CSF barrier (5). During inflammatory conditions, normal T-cells can migrate to the site of infection using a range of different mechanisms, including chemokine gradients and cytoskeletal remodeling (9, 10). T-cell motility is also regulated by integrin-receptor binding, where LFA1 is a key integrin that binds to the adhesion molecule ICAM1 (11, 12). Activation of LFA1 is induced through a pathway called inside-out signaling, initiated by the T-cell receptor (TCR) (11, 12). After TCR activation, kinase recruitment drives the formation of the LAT signalosome, triggering adaptor-mediated RAP1 activation that induces conformational changes in LFA1, enabling binding to ICAM1 and thereby regulation of T-cell migration (13). ICAM1 is upregulated in the CNS to increase normal T-cell recruitment during immune reactions (14, 15). However, how this pathway is regulated in malignant conditions remains unclear. In T-ALL, leukemic blasts are arrested at different stages of thymocyte development, resulting in heterogeneous immunophenotypes and variable TCR expression. Whether the inside-out signaling pathway is hijacked by T-ALL during CNS infiltration, through the TCR or alternative mechanisms, remains to be determined.

To determine whether alterations in the TCR and subsequent signaling events are characteristic of CNS-infiltrated T-ALL, we analyzed RNA-sequencing data from pediatric T-ALL patients (generated by the TARGET initiative). Functional enrichment analysis of CNS-infiltrated and CNS-relapsed T-ALL samples revealed dysregulation of the TCR complex as well as upregulation of adaptor protein SKAP1 (also known as SKAP55), which plays a central role in inside-out signaling. Furthermore, T-ALL cells were co-cultured with CNS-derived meningeal cells and exposed to MTX, which resulted in alterations to inside-out signaling and the transcription factor NFATC1. Knockdown of SKAP1 in T-ALL cells lead to a loss of cell proliferation and cell viability. Together, our results suggest an association between SKAP1, inside-out signaling and CNS infiltration in pediatric T-ALL.

## RESULTS

### CNS-infiltrated and CNS-relapsed T-ALL patients were distinguished by a dysregulation of genes involved in extracellular matrix organization and degradation.

**Figure 1:**
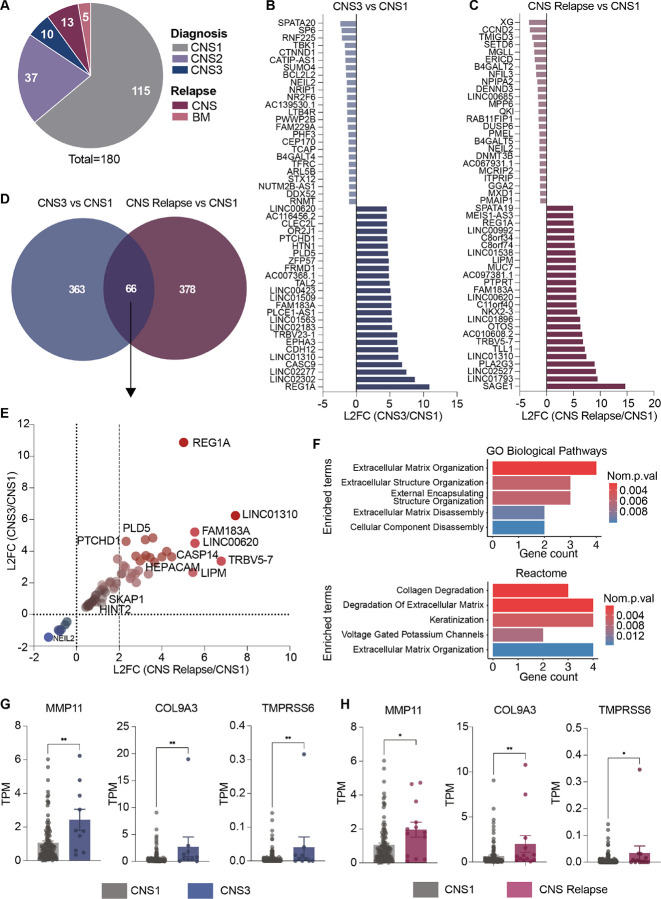
Differential gene expression in CNS-infiltrated and CNS-relapsed T-ALL compared to non-CNS-infiltrated T-ALL. **A)**Clinical characteristics of included pediatric T-ALL samples (total n=180). Samples from time of diagnosis were subdivided based on the clinical characterization of CNS infiltration, into CNS1 (n=115), CNS2 (n=37) or CNS3 (n=10). Samples taken at relapse were subdivided by site of relapse; CNS (n=13) or BM (n=5). **B)**Top differentially expressed genes (p<0.05) in CNS3 (n=10) samples compared to CNS1 (n=115) samples. Data displayed as L2FC. Significance calculated by unpaired students t-test. **C)**Top differentially expressed genes (p<0.05) in CNS relapse (n=13) samples compared to CNS1 (n=115) samples. Data displayed as L2FC. Significance calculated by two-tailed unpaired students t-test. **D)**Venn diagram of significantly differentially expressed genes (p<0.05) in CNS3 (n=10) and CNS relapse (n=13) compared to CNS1 (n=115). Significance calculated by two-tailed unpaired students t-test. **E)**Volcano plot of all common differentially expressed genes (p<0.05, total of 66 genes) in CNS3 (n=10) and CNS relapse (n=13) compared to CNS1 (n=115). Data displayed as L2FC in CNS3 (y-axis) and CNS relapse (x-axis) compared to CNS1. Significance calculated by two-tailed unpaired students t-test. **F)**Functional enrichment analysis of the commonly differentially expressed genes (total of 66 genes) in CNS3 (n=10) and CNS relapse (n=13) compared to CNS1 (n=115) using EnrichR (v3.2) in R (version 4.3.1), with GO biological processes (upper graph) and Reactome (lower graph) gene sets. Significance is displayed as nominal p-value. **G)**Expression of MMP11, COL9A3 and TMPRSS6 displayed as TPM in CNS3 (n=10) compared to CNS1 (n=115) samples. Significance calculated by two-tailed unpaired students t-test (*p<0.05, **p<0.01). **H)**Expression levels of MMP11, COL9A3 and TMPRSS6 displayed as TPM in CNS relapse (n=13) samples compared to CNS1 (n=115) samples. Significance calculated by two-tailed unpaired students t-test (*p<0.05, **p<0.01).

To identify genetic alterations that characterize CNS-infiltrated and CNS-relapsed T-ALL, RNA-sequencing analysis was performed on 180 pediatric bone marrow (BM)-derived T-ALL samples, collected at diagnosis and relapse (non-longitudinal) (Supplementary Table S2). Given the limited availability and invasive nature of cerebrospinal fluid (CSF) samples in pediatric patients, BM-derived samples were utilized, which may provide a clinically relevant and accessible source to identify molecular features associated with CNS disease. Diagnosis samples were divided based on the clinical classification of CNS infiltration into CNS1, CNS2 or CNS3 (Fig. 1A, SF1A). Relapse samples were grouped based on the site of relapse; either in the CNS or the BM (Fig. 1A, SF1A). We focused on the subgroups with advanced CNS infiltration (CNS3, n = 10) and patients who relapsed in the CNS (CNS relapse, n = 13) compared with patients without CNS infiltration (CNS1, n = 115). Analysis of overall gene expression revealed that most genes were expressed at a lower level in both CNS3 and CNS relapse samples compared to CNS1 samples (Figure SF1B-C). However, among the significantly differentially expressed genes (approximately 2.2% of all genes), most were upregulated in both CNS3 (74%) and CNS relapse (85%) (Figure SF1B, SF1C). TAL2, a transcription factor closely related to T-ALL oncogene TAL1, was upregulated in CNS3 patients compared to CNS1 patients (Fig. 1B) (16, 17). CNS relapse patients were characterized by downregulation of Cyclin D2 (CCND2), a known cell cycle regulator (Fig. 1C) (18).

In both CNS3 and CNS relapse samples, more than 400 genes were differentially expressed compared to CNS1 samples, with 66 genes commonly dysregulated between both groups (Fig. 1D–E, SF1D). Among these genes we identified an upregulation of HINT2, which controls mitochondria-induced apoptosis, as well as HEPACAM, an adhesion molecule linked to cell cycle arrest in breast cancer cells (Fig. 1E, SF1E-F) (19, 20). The commonly dysregulated genes were analyzed using EnrichR (21), which revealed that the differentially expressed genes were associated with extracellular matrix (ECM) organization and degradation in both the Reactome and Gene Ontology (GO) Biological Processes (BP) libraries (Fig. 1F). Notable genes involved in these pathways included COL9A3, TMPRSS6 and metalloproteinase MMP11, which were all upregulated in CNS3 and CNS relapse compared with CNS1 samples (Fig. 1G–H). Upregulation of genes involved in ECM remodeling and degradation, such as MMPs, is consistent with processes that could facilitate leukemia cell migration to the CNS (22).

### Alterations to the TCR complex and downstream signaling via SKAP1 in CNS-infiltrated and CNS-relapsed T-ALL.

**Figure 2:**
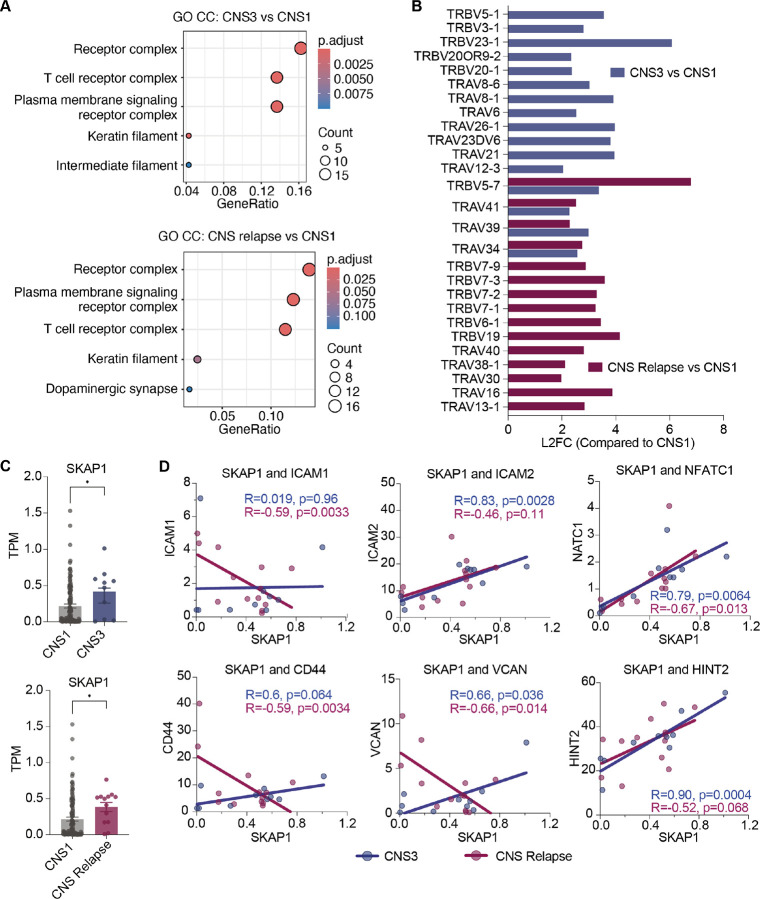
Dysregulation of the TCR complex in CNS-infiltrated and CNS-relapsed T-ALL. **A)**ORA of differentially expressed genes (p<0.05, L2FC>2) in CNS3 (n=10) compared to CNS1 (n=115) samples (upper graph) and CNS relapse (n=13) compared to CNS1 (n=115) samples (lower graph), performed in R (version 4.3.1) using the GO cellular compartment (CC) gene set. Significance displayed as adjusted p-value. **B)**Dysregulated genes (p<0.05) from variable segments of the TCR complex in CNS3 (n=10) and CNS relapse (n=13) compared to CNS1 (n=115). Data displayed as L2FC, significance calculated by two-tailed unpaired students t-test. **C)**Expression of SKAP1 displayed as TPM in CNS3 (n=10) compared to CNS1 (n=115) samples (upper graph) and CNS relapse (n=13) compared to CNS1 (n=115) samples (lower graph). Significance calculated by two-tailed unpaired students t-test (*p<0.05). **D)**Correlations of SKAP1 and ICAM1, ICAM2, NFATC1, CD44, VCAN and HINT2 in CNS3 (n=10) and CNS relapse (n=13) samples separately (CNS3 in blue, CNS relapse in purple). Correlation was calculated using Pearson correlation coefficients, two-tailed with 95% confidence interval. Data displayed as TPM values (x-axis and y-axis) and results shown as the R value and p-value of each group.

To further characterize CNS-infiltrated T-ALL, over-representation analysis (ORA) was performed using all significantly differentially expressed genes in CNS3 and CNS relapse compared to CNS1, respectively. Analysis of GOs revealed an enrichment of genes in Cellular Compartments (CC) related to the TCR complex in both CNS3 and CNS relapse samples (Fig. 2A, SF2A-B). Many genes coding for variable regions of the α and β chain of the TCR were found to be upregulated in CNS-infiltrated and CNS-relapsed T-ALL (Fig. 2B). Moreover, SKAP1, crucial for TCR-mediated inside-out signaling was upregulated in both CNS3 and CNS relapse samples (Fig. 2C). SKAP1 was one of the 66 genes commonly altered in CNS-implicated T-ALL compared to CNS-negative samples (Fig. 1D). Other components of inside-out singling, such as ADAP1, RAP1 (RAP1A and RAP1B) and SKAP1-paralogue SKAP2, were not altered in CNS3 or CNS relapse patients (Figure SF2C).

Subsequently, we explored correlations between SKAP1, and other genes related to TCR-signaling and adhesion in the T-ALL dataset. In CNS3 patients, nearly all substantial correlations (R > 0.6 or R<−0.06) were positive (96%), while CNS relapse samples showed a larger variety of associations (60% positive, 40% negative) (Figure SF2D-E). CNS relapse patients displayed a negative correlation between SKAP1 and adhesion genes ICAM1 and CD44, while CNS3 patients showed no correlation, or a positive correlation (Fig. 2D). Both CNS3 and CNS relapse samples displayed a positive correlation between SKAP1 and ICAM2, which like ICAM1, binds to LFA1, but has a more controversial role in tumor cells (Fig. 2D) (23). Opposing correlations (positive in CNS3 and negative in CNS relapse) were observed between SKAP1 and VCAN, which is associated with epithelial-mesenchymal transition (EMT) (Fig. 2D) (24). In both groups, a positive correlation was identified between SKAP1 and NFATC1, a transcription factor downstream of the TCR, as well as between SKAP1 and HINT2 (Fig. 2D) (19, 25). Although functional effects of SKAP1 in CNS-infiltrated T-ALL requires further investigation, our data indicates both similar and divergent relationships between SKAP1 expression and genes involved in adhesion and migration in CNS3 and CNS relapse samples.

### T-ALL cells exhibit dysregulation of targets downstream of the inside-out signaling pathway compared to normal HSPCs.

**Figure 3:**
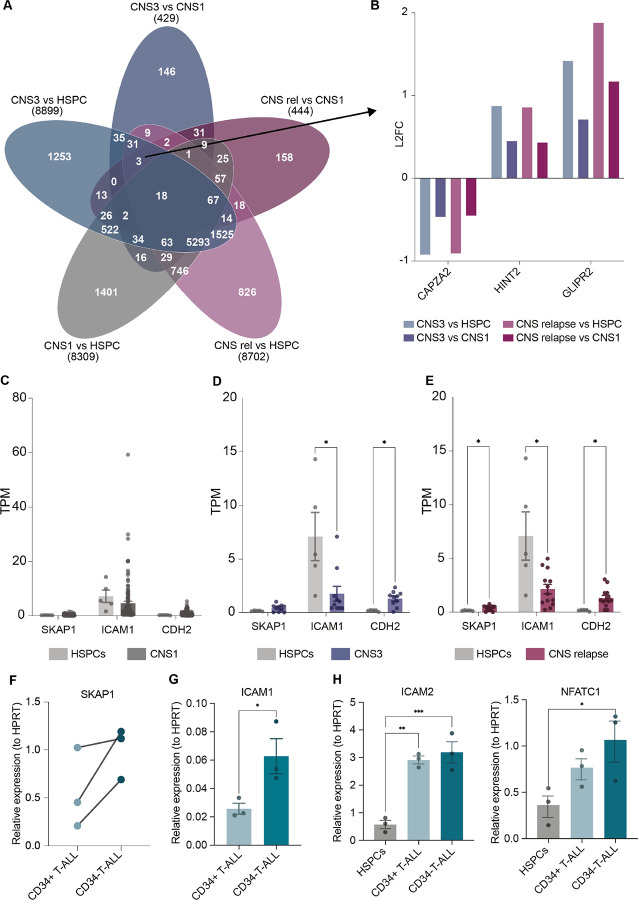
T-ALL cells exhibit dysregulation of targets downstream of the inside-out signaling pathway compared to HSPCs. **A)**Venn diagram of differentially expressed genes (p<0.05) in T-ALL (CNS1, CNS3 and CNS relapse separately) compared to normal HSPCs (CD34+ CB, n=5) as well as within the T-ALL samples (CNS3 (n=10) and CNS relapse (n=13) compared to CNS1 (n=115)). Significance calculated by two-tailed unpaired students t-test. **B)**Genes (CAPZA2, HINT2 and GLIPR2) significantly altered (p<0.05) in both CNS3 (n=10) and CNS relapse (n=13) compared to both CNS1 (n=115) and HSPCs (n=5). Significance calculated by two-tailed unpaired students t-test. **C)**Expression of SKAP1, ICAM1 and CDH2 (N-cadherin) in CNS1 (n=115) T-ALL samples compared to normal HSPCs (n=5), separately. Significance calculated by multiple two-tailed, unpaired student’s t-test with Holm-Šídák correction for multiple comparisons, displayed as adjusted p-value. **D)**Expression of SKAP1, ICAM1 and CDH2 (N-cadherin) in CNS3 (n=10) T-ALL samples compared to normal HSPCs (n=5), separately. Significance calculated by multiple two-tailed, unpaired student’s t-test with Holm-Šídák correction for multiple comparisons, displayed as adjusted p-value (*p<0.05). **E)**Expression of SKAP1, ICAM1 and CDH2 (N-cadherin) in CNS relapse (n=13) T-ALL samples compared to normal HSPCs (n=5), separately. Significance calculated by multiple two-tailed, unpaired student’s t-test with Holm-Šídák correction for multiple comparisons, displayed as adjusted p-value (*p<0.05). **F)**Mean expression of SKAP1 in LICs (CD34+, n=3) and blast cells (CD34-, n=3) from primary pediatric T-ALL patients (matched samples). Data displayed as relative expression compared to housekeeping gene (HPRT). **G)**Expression of ICAM1 in LICs (CD34+, n=3) and blast cells (CD34-, n=3) from primary pediatric T-ALL patients (matched samples). Data displayed as relative expression compared to housekeeping gene (HPRT). Significance calculated by two-tailed paired students t-test (*p<0.05). **H)**Expression of ICAM2 and NFATC1 in LICs (CD34+, n=3) and blast cells (CD34-, n=3) from primary pediatric T-ALL patients (matched samples) compared to normal HSPCs (CD34+ CB, n=3). Data displayed as relative expression compared to housekeeping gene (HPRT). Significance calculated by one-way ANOVA comparing each group to HSPCs, displayed as adjusted p-value by Dunnett’s correction (*p<0.05, **p<0.01, ***p<0.001).

To further investigate the role of inside-out signaling in CNS-infiltrated T-ALL, we compared the T-ALL dataset with healthy hematopoietic stem and progenitor cells (HSPCs, CD34 + CB, n = 5). By comparing differentially expressed genes in CNS3 and CNS relapse samples to CNS1 samples and HSPCs, we identified genes specifically altered in CNS-implicated T-ALL (Fig. 3A, Supplementary table S3). Only three genes were significantly altered in CNS3 and CNS relapse samples compared to both CNS1 samples and HSPCs: CAPZA2, GLIPR2 and previously identified HINT2 (Fig. 3B). CAPZA2 has been reported to regulate actin cytoskeleton stability, and GLIPR2 has been associated with EMT and migration by activating the ERK1/2 cascade (26, 27).

While SKAP1 expression did not differ between CNS1 samples and HSPCs, SKAP1 levels were higher (although not significant) in CNS3 samples and significantly upregulated in CNS relapse samples (Fig. 3C–E). In contrast, ICAM1 was downregulated in both CNS3 and CNS relapse samples compared with HSPCs (Fig. 3C–E). Moreover, a loss of SKAP2 was identified in all T-ALL samples compared with HSPCs, along with reduced expression of ADAP1 and RAP1A in CNS-implicated samples, while no significant difference was found in RAP1B (Figure SF3A-C). RNA-sequencing analysis also identified upregulation of CDH2, known as N-cadherin, a key regulator of EMT, in both CNS3 and CNS relapse samples, but not in CNS1 samples, compared to HSPCs (Fig. 3C–E) (28). These data strengthen the findings in CNS-infiltrated T-ALL, highlighting alterations that are restricted to CNS3 and CNS relapse samples and not observed in CNS negative samples.

Although it remains unclear which T-ALL cells acquire the capacity to infiltrate the CNS, detection of blast cells in the CSF currently forms the basis for diagnosis (4). To link CNS-associated differences in T-ALL with characteristics specific to T-ALL blasts, we utilized primary pediatric T-ALL samples. From peripheral blood mononuclear cells (PBMCs), we isolated the leukemia initiating cell (LIC) enriched fraction of cells (CD34+) and compared to the remaining blast cells (CD34-) and HSPCs. RT-qPCR analysis indicated a higher mean expression of SKAP1 in the blast population of cells, compared to the matched LICs (Fig. 3F). The adhesion molecule ICAM1, downstream of SKAP1, was upregulated in blast cells compared to LICs (Fig. 3G). While there was no significant difference in ICAM1 expression between the two T-ALL cell populations compared with HSPCs, we found upregulation of ICAM2 in both blasts and LICs (Fig. 3H, SF3D). Additionally, blast cells expressed higher levels of the transcription factor NFATC1 and the EMT-associated gene VCAN compared with normal HSPCs (Fig. 3H, SF3D).

### Knockdown of SKAP1 in T-ALL cells reduced cell proliferation and viability.

**Figure 4:**
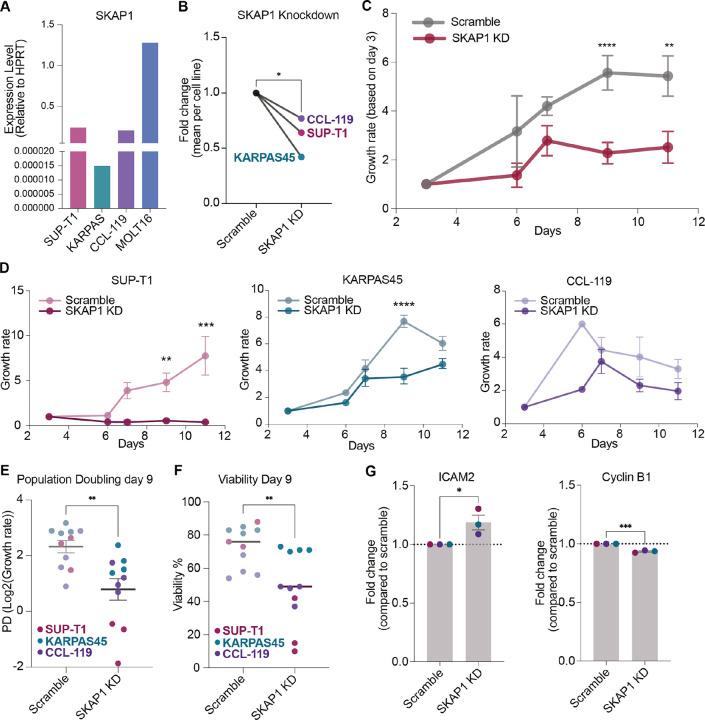
Knockdown of SKAP1 in T-ALL cells reduced cell proliferation and viability. **A)**Expression of SKAP1 (relative to HPRT) in T-ALL cell lines SUP-T1, KARPAS45, CCL-119 and MOLT16 (n=1 per cell line). **B)**Lentiviral knockdown of SKAP1 compared to scramble control in T-ALL cell lines SUP-T1, KARPAS45 and CCL-119. Data displayed as fold change compared to the scramble control (mean per cell line of replicated experiments). Significance calculated by two-tailed unpaired students t-test (*p<0.05). **C)**Growth rate (fold change compared to day 3 post-transduction) of T-ALL cell lines SUP-T1, KARPAS45, CCL-119 (n=3–11 per time-point) from day 3 to day 11 post transduction, in SKAP1 knockdown compared to the scramble control. Significance was calculated using two-way ANOVA with Šídák's multiple comparisons correction (**p<0.01, ****p<0.0001). **D)**Growth rate (fold change compared to day 3 post-transduction) of T-ALL cell lines SUP-T1 (n=1–3 per time-point), KARPAS45 (n=1–4 per time-point), CCL-119 (n=1–4 per time-point) separately, from day 3 to day 11 post transduction, in SKAP1 knockdown compared to the scramble control. Significance was calculated using two-way ANOVA with Šídák's multiple comparisons correction (**p<0.01, ***p<0.001, ****p<0.0001). **E)**Population doubling (log2 growth rate based on day 3) on day 9 post-transduction of T-ALL cell lines SUP-T1 (pink, n=3), KARPAS45 (green, n=4), CCL-119 (purple, n=4). Significance calculated by two-tailed unpaired students t-test (**p<0.01). **F)**Viability (percentage) on day 9 post-transduction of T-ALL cell lines SUP-T1 (pink, n=3), KARPAS45 (green, n=4), CCL-119 (purple, n=4). Significance calculated by two-tailed unpaired students t-test (**p<0.01). **G)**Expression of ICAM2 and Cyclin B1 (CCNB1) in T-ALL cell lines SUP-T1, KARPAS45 and CCL-119 after SKAP1 knockdown compared to the scramble control. Data displayed as fold change compared to the scramble control (n=3 per condition, cell lines displayed as biological replicates). Significance calculated by two-tailed unpaired students t-test (*p<0.05, ***p<0.001).

To assess the functional effects of SKAP1 and subsequent signaling events, four different T-ALL cell lines (SUP-T1, KARPAS45, CCL-119 and MOLT16), with varying endogenous SKAP1 expression, were utilized for lentiviral knockdown (Fig. 4A). The included T-ALL cell lines displayed different culture patterns, with SUP-T1 and KARPAS45 cells growing in clusters, while CCL-119 and MOL16 cells grew in single-cell suspension (Figure SF4A). The cells were transduced with shRNA targeting SKAP1 or a scramble control (MOLT16 was excluded due to insufficient knockdown) (Fig. 4B, SF4B). T-ALL cells with diminished SKAP1 expression (cell lines displayed as biological replicates) displayed a significantly lower growth rate compared with the scramble control (Fig. 4C). Notably, cells that grow in clusters under normal conditions (SUP-T1 and KARPAS45) demonstrated a more apparent inhibition of cell growth (Fig. 4D). The cells were observed for up to 11 days post-transduction and the most substantial loss of both population doubling and cell viability was observed at day 9 (Fig. 4E–F). Furthermore, RT-qPCR analysis revealed that knockdown of SKAP1 caused upregulation of ICAM2 and a modest reduction of cell cycle regulator Cyclin B1 (CCNB1) (29), strengthening our findings regarding the pro-proliferative capacities of SKAP1 in T-ALL (Fig. 4G).

### T-ALL cells exposed to MTX displayed a loss of inside-out signaling when grown in co-culture with HMCs.

**Figure 5:**
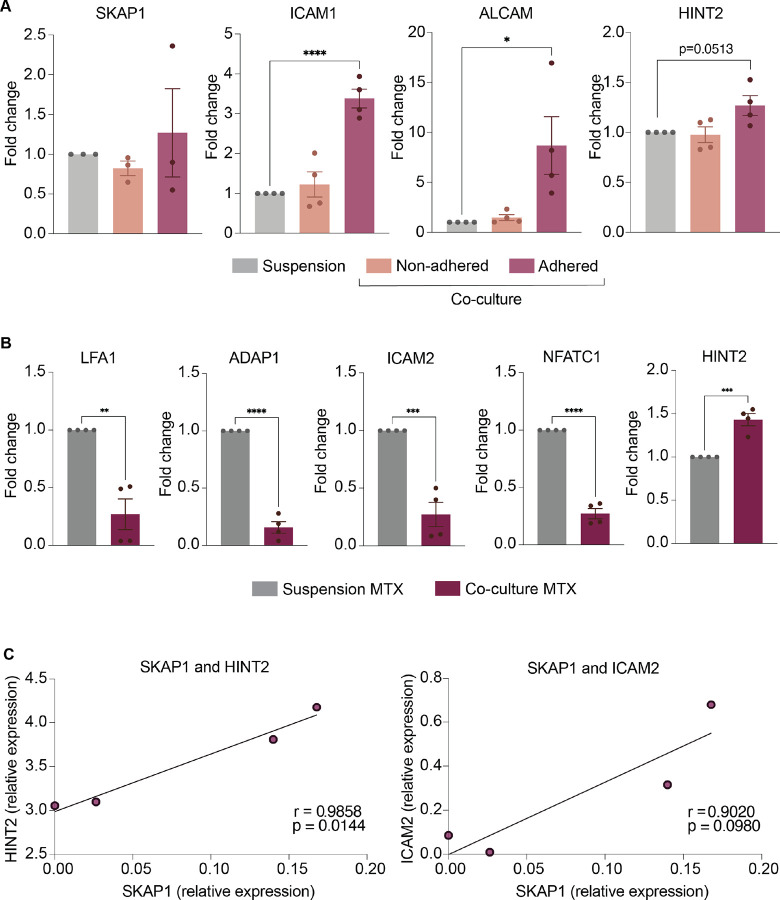
Alterations to inside-out signaling in MTX-treated T-ALL cells in HMC co-culture. **A)**Expression of SKAP1, ICAM1, ALCAM and HINT2 in T-ALL cell lines SUP-T1, KARPAS45, CCL-119 and MOLT16 grown in HMC co-culture (adhered and non-adhered) compared to T-ALL cells grown alone in suspension. Results displayed as fold change of T-ALL cells (biological replicates n=4 per condition, except for SKAP1 with n=3 due to undetectable expression in KARPAS45) in co-culture compared to their respective control in suspension. Significance was calculated using ordinary one-way ANOVA with multiple comparisons compared to suspension, using Dunnett correction (*p<0.05, ****p<0.0001). **B)**Expression of LFA1, ADAP1, ICAM2, NFATC1 and HINT2 in T-ALL cell lines SUP-T1, KARPAS45, CCL-119 and MOLT1 grown in HMC co-culture (displayed as biological replicates, n=4 per condition) compared to T-ALL cells grown alone in suspension after exposure to 2uM MTX. Results displayed as fold change of T-ALL cells in co-culture compared to their respective control in suspension. Significance calculated by two-tailed unpaired students t-test (**p<0.01, ***p<0.001, ****p<0.0001). **C)** Correlation of SKAP1 and HINT2 as well as SKAP1 and ICAM2 in T-ALL cell lines SUP-T1, KARPAS45, CCL-119 and MOLT1 grown in HMC co-culture after exposure to 2uM MTX (displayed as biological replicates, n=4 per condition). Data displayed as relative expression compared to the housekeeping gene (x-axis and y-axis) and results shown as the R value and p-value of each group. Correlation was calculated using Pearson correlation coefficients, two-tailed with 95% confidence interval.

To analyze direct effects on inside-out signaling in T-ALL cells interacting with cells from the CNS niche, T-ALL cell lines (SUP-T1, KARPAS45, CCL-119 and MOLT16) were cultured together with CNS-derived meningeal cells in a co-culture model, or alone in suspension. HMC co-culture did not significantly affect the expression of key components of inside-out signaling, including SKAP1, ADAP, LFA1, NFATC1 or ICAM2 (Fig. 5A, SF5A). However, the LFA1 ligand ICAM1 and adhesion molecule ALCAM were upregulated in T-ALL cells adhered to HMCs in the co-culture (Fig. 5A). T-ALL cells in co-culture showed an indication of a higher expression of HINT2, which was found to be upregulated in CNS-infiltrated T-ALL samples (Fig. 5A).

Next, T-ALL cells were treated with 2 μM methotrexate (MTX), a chemotherapeutic agent routinely used in pediatric T-ALL, including CNS-directed treatment, to assess treatment-induced transcriptomic effects. When exposed to MTX, T-ALL cells in co-culture underwent changes in several components of the inside-out signaling pathway, including loss of LFA1 and ADAP1, compared to cells in suspension (Fig. 5B). However, no significant alterations in SKAP1 expression were observed (Figure SF5B). MTX treatment of T-ALL cells led to a downregulation of ICAM2, but not ICAM1 when grown in co-culture (Fig. 5B, SF5B). Cells in MTX-treated co-culture also revealed a downregulation of NFATC1 (Fig. 5B). Similar to CNS-infiltrated T-ALL samples from the RNA-sequencing analysis, HINT2 was upregulated in T-ALL cells grown in co-culture (Fig. 5B). Moreover, a positive correlation was identified between SKAP1 and HINT2, and between SKAP1 and ICAM2 in co-cultured T-ALL cells exposed to MTX (Fig. 5C). Our analysis identified alterations in multiple genes involved in inside-out signaling in T-ALL cells in MTX-treated co-culture, indicating a possible role for these signaling events in CNS-infiltrated T-ALL, but this requires further analysis.

## DISCUSSION

To improve our understanding of CNS-infiltrated T-ALL, we investigated possible transcriptional alterations that could be characteristic for patients with CNS disease. We identified dysregulation of ECM organization, the TCR complex and SKAP1-mediated inside-out signaling in both CNS-infiltrated and CNS-relapsed pediatric T-ALL. Knocking down SKAP1 led to reduced proliferation and viability of T-ALL cells. Moreover, T-ALL cells co-cultured with meningeal cells displayed loss of components of the inside-out signaling pathway when exposed to MTX. Together, these findings highlight TCR-mediated signaling and adhesion-related programs as transcriptional features associated with CNS infiltration in pediatric T-ALL.

To predict and prevent CNS infiltration and CNS-originated relapse we need to understand how leukemia cells invade and persist in the CNS. One factor controlling metastasis is the ECM, which must be modulated to enable infiltration of cells into other tissues (30–32). In this study we identified an enrichment of genes involved in ECM organization and degradation, including COL9A3, which has been suggested to regulate blood-CSF permeability through the SOX9-COL9A3 axis, and MMP11 that has been associated with facilitating tumor cell migration (22, 33, 34). Another possible mechanism that could be utilized by leukemia cells is integrin-mediated adhesion, regulated through the TCR and the inside-out signaling pathway, resulting in LFA1-ICAM1 binding (11, 12, 35). Our study revealed an enrichment of the TCR complex and downstream adaptor protein SKAP1 in both CNS-infiltrated and CNS-relapsed T-ALL compared with CNS negative patients. Notably, CNS-implicated samples exhibited an upregulation of migration-associated genes, such as SKAP1 and N-cadherin, compared with normal HSPCs. These changes were absent in CNS negative samples, indicating CNS-specific features rather than general disease-associated alterations. Furthermore, NFATC1 was upregulated in primary T-ALL blast cells compared with HSPCs. NFATC1 has been linked to promoting the invasive capacity of malignant cells across multiple tumor types (4, 36–39). Together, our results highlight transcriptional alterations in ECM-regulatory genes, and several adhesion and migration associated genes, in CNS-infiltrated and CNS-relapsed T-ALL. These changes may reflect adaptations linked to CNS infiltration and interaction with the CNS microenvironment, although their functional relevance requires further validation in advanced in vivo models.

We have previously shown that direct interactions between T-ALL cells and HMCs in a CNS co-culture model disrupted cell cycle regulation and induced expression of adhesion molecules (40). We utilized the same co-culture model to explore inside-out signaling. Although no significant differences were found in SKAP1 expression, an upregulation of ICAM1 was identified in T-ALL cells adhered to meningeal cells. Interestingly, when T-ALL cells were exposed to MTX, a standard component of CNS-directed treatment, there was an overall reduction in components of the inside-out signaling pathway, except for SKAP1, which remained unchanged. Whether these effects are specific to chemotherapy exposure requires further investigation; however, these findings suggest that MTX treatment may be associated with alterations in inside-out signaling in T-ALL cells interacting with meningeal cells.

The specific role of SKAP1 in malignant development remains poorly defined. Overexpression of SKAP1 has been reported in several cancers, including gastric and colon cancer, where it has been associated with tumor progression and immune evasion (41, 42). Given its prominent role in T-cell biology, aberrant SKAP1 expression may be particularly relevant in T-cell malignancies, such as T-ALL (43). To address this, we knocked down SKAP1 in T-ALL cell lines, which led to reduced proliferation and viability. Loss of SKAP1 caused a modest downregulation of Cyclin B1, a key regulator of the G2/M transition (29). Although SKAP1 is not known to directly interact with cyclins or CDKs, it may influence cell-cycle progression through upstream regulators such as PLK1, which can control G2/M transition and mitotic entry (44). Moreover, SKAP1 knockdown in T-ALL cells led to increased ICAM2 expression, which has primarily been recognized for having tumor suppressive effects (23, 45, 46). In contrast, ICAM2 was found to promote migration across the blood–CSF barrier and leptomeningeal metastasis, in triple-negative breast cancer (47).

Our findings point to SKAP1-mediated inside-out signaling as part of transcriptional programs associated with cell proliferation and CNS infiltration in pediatric T-ALL. Canonically, inside-out signaling is initiated downstream of the TCR (12). However, T-ALL arises from thymocytes arrested at distinct developmental stages, resulting in heterogeneous CD3 and TCR surface expression (48). While some T-ALL subtypes express a fully assembled αβ TCR, others express an incompletely assembled pre-TCR or lack surface TCR expression altogether (49). This developmental heterogeneity raises the possibility that integrin activation may not be regulated uniformly across T-ALL subtypes. Notably, we observed an enrichment of SKAP1 in CNS-implicated T-ALL, independent of direct TCR assessment. This suggests that SKAP1 and inside-out signaling may not strictly require a fully mature surface TCR. SKAP1 expression may be retained even in developmentally arrested leukemias, either as part of a broader T-lineage program or through activation by alternative oncogenic pathways. Whether SKAP1 activation in T-ALL remains dependent on TCR engagement or reflects TCR-independent signaling mechanisms warrants further functional investigation.

A limitation of this study is the use of BM-derived samples, which may not fully capture the molecular landscape of leukemia cells within the CNS microenvironment. However, given the challenges associated with obtaining CSF samples from pediatric patients, BM represents a practical and clinically relevant source of material. Importantly, our findings demonstrate that BM-derived leukemia cells harbor specific transcriptomic signatures characteristic for patients diagnosed with CNS infiltration, suggesting that systemic leukemic populations may reflect, at least in part, CNS-related biology. This highlights the potential of using more accessible BM samples to identify markers of CNS leukemia, but future studies integrating matched BM and CSF samples will be important to further validate these findings.

In conclusion, our findings identify SKAP1 and inside-out signaling as potential features associated with adhesion and proliferation in CNS-infiltrated pediatric T-ALL. Transcriptomic enrichment of extracellular matrix and TCR-mediated signaling programs appears characteristic of CNS-infiltrated and CNS-relapsed pediatric T-ALL. Together, these findings provide new insight into transcriptional programs associated with CNS infiltration in T-ALL and suggest that inside-out signaling may represent a potential therapeutic or diagnostic target.

## METHODS

### Downloading and normalization of publicly available RNA-sequencing data

RNA-sequencing data from pediatric T-ALL patients (n = 180) was publicly available by the TARGET initiative. The RNA-sequencing dataset (.BAM) was acquired via the GDC Data Portal (https://portal.gdc.cancer.gov/projects/TARGET-ALL-P2). A total of 180 BM-derived T-ALL samples were included, 162 from the time of diagnosis and 18 from relapsed patients (not longitudinal). Diagnosis samples were subdivided by the clinical characterization for CNS infiltration, into CNS1 (n = 115), CNS2 (n = 37), or CNS3 (n = 10). Relapse samples were grouped depending on the site of relapse, either BM (n = 5) or CNS (n = 13). Raw counts were generated using the SeqMonk Mapped Sequence Data Analyzer tool (v1.47.1) with the pipeline described in Enlund et.al (40). RNA-sequencing from CD34^+^ cord blood was obtained from a publicly available data set (GSE190269) from the Gene Expression Omnibus (50). Raw counts were normalized in R (v4.3.1) to transcripts per million (TPM) following the GDC bioinformatics pipeline. Non-coding and novel genes were excluded from the analysis.

### Functional enrichment analysis

Functional gene set enrichment analysis and ORA were performed using R (v4.3.1) with the packages EnrichR (v3.2), clusterProfiler (v4.8.3) and enrichplot (v1.20.3). EnrichR analysis included common differentially expressed genes in CNS3 (n = 10) and CNS relapse (n = 123) vs CNS1 (n = 115) samples. ORA included all differentially expressed genes with a L2FC above 2 in CNS3 and CNS relapse vs CNS1 samples, respectively.

### Cell culture

T-ALL cell lines CCL-119 and SUP-T1 (ATCC, Manassas, VA, USA) were cultured in RPMI (Sigma-Aldrich, St. Louis, MO, USA) supplemented with 10% FBS (Sigma-Aldrich), MOLT16 (Leibniz Institute DSMZ, Braunschweig, Germany) was cultured in IMDM (Gibco, Thermo Fisher Scientific, Grand Island, NY, USA) supplemented with 10% FBS and KARPAS-45 was cultured in RPMI (Sigma-Aldrich), supplemented with 2 mM glutamine and 20% FBS (Sigma-Aldrich). Immortalized Human Meningeal Cells (HMCs) (Innoprot, Bizkaia, Spain) were cultured in complete Meningeal Cell Medium (Innoprot) and detached using the Primary Cells Detach Kit (Innoprot).

### Primary patient samples

Peripheral blood mononuclear cells from primary T-ALL patient samples (n = 3) were obtained from patients at Karolinska University Hospital Pediatric Oncology Ward (Ethical permission: dnr 2023-07390-02) after oral and written informed consent. PBMCs from primary T-ALL samples underwent CD34 positive selection using EasySep human CD34 positive selection kit II (STEMCELL Technologies, Vancouver, British Columbia, Canada). Cord blood (CD34+, n = 3) was purchased from BioNordika (Stockholm, Sweden).

### Reverse transcription and RT-qPCR

RNA preparation (Qiagen, Hilden, Germany), reverse transcription (Invitrogen, Carlsbad, CA, USA) and RT-qPCR analysis (Invitrogen) were performed according to provided protocols and as described in Enlund et al (40). Primer sequences can be found in supplementary table S1.

### Lentiviral knockdown

Human shRNA lentiviral particles targeting SKAP1 and a scramble control (TL309635V, OriGene Technologies, Rockville, MD, USA) were used to knockdown SKAP1 in T-ALL cell lines. Each cell line represents a biological replicate, n = 3 per condition (MOLT16 was excluded due to insufficient knockdown), with minimum of two technical replicates per cell line. Lentiviral transduction was performed at a multiplicity of infection (MOI) of 20 to 50 and cells were harvested 72 hours post-transduction. Viability and live cell count were quantified by trypan blue staining (Bio-Rad, Hercules, CA, USA) and knockdown efficacy was determined by RT-qPCR.

### Co-culture

To determine the effects of interactions between T-ALL cells and meningeal cells, we utilized a previously established CNS co-culture model, as described in Enlund et.al (40). T-ALL cell lines were cultured either alone in suspension or together with IM-HMCs (Innoprot) at a ratio of 3:1 leukemia cells to IM-HMCs. Each cell line represents a biological replicate, n = 4 per condition, with minimum of two technical replicates per cell line. Non-adherent cells were collected after 24 hours followed and adhered cells were harvested and separated from IM-HMCs using EasySep Human CD3 positive Selection kit II (Stem Cell Technologies) after an additional 48 hours. In conditions where cells were treated with chemotherapy, 2uM of MTX (Sigma-Aldrich) was added after 24 hours. Viability and cell count were determined using tryphan blue staining (Bio-Rad).

### Statistical analysis

Statistical analyses were performed using unpaired two-tailed students t-test, one-way ANOVA with Dunnett’s correction or two-way ANOVA with Šídák's multiple comparisons correction. Correlation was determined by Pearson correlation coefficients, two-tailed with 95% confidence interval. Analyses were performed using GraphPad Prism (v10) or R (version 4.3.1). Results are presented as the mean ± SEM. Significance was set to p-value < 0.05.

## Supplementary Files

This is a list of supplementary files associated with this preprint. Click to download.

- CNSTCRsupplemental1.png
- CNSTCRSupplemental2.png
- CNSTCRsupplemental3.png
- CNsTCRsupplemental4.png
- CNSTCRsupplemental5.png
- Supplementalfigurelegends.docx
- Supplementaltable1.xlsx
- Supplementaltable2.xlsx
- Supplementaltable3.xlsx

## Data Availability

The RNA-sequencing dataset used in this study is based upon data generated by the Therapeutically Applicable Research to Generate Effective Treatments (TARGET) initiative, phs000218, managed by the NCI. The data used for this analysis are available at https://portal.gdc.cancer.gov/projects/TARGET-ALL-P2. Further information and requests for resources and reagents should be directed to and will be fulfilled by Dr. Frida Holm (frida.holm@ki.se).
